# Old Point Comfort

**Published:** 1897-09

**Authors:** 


### Editorial.

### OLD POINT COMFORT.

The meeting of the American Dental Association at Old Point Comfort was anticipated with mingled feelings of hope and anxiety. When the sessions closed in 1896 at Saratoga Springs, the hope was expressed that the meeting in 1897 would result in a union of all the professional elements—North, East, South, and West—in an organization which should truly represent the national feeling in the various sections of the country. This hope grew, as the year passed away, into a conviction that this could be accomplished, notwithstanding much opposition expressed in certain quarters.

The two conventions, the American and the Southern, met separately at Old Point Comfort, the one at the Chamberlain and the other at the Hygeia. The Joint Committee of both bodies had, during the year preceding, prepared a course of action for both organizations, thus preventing delay and, perhaps, much friction.

The work of both Associations proceeded as usual; that of the American was excellent in a scientific sense, suffering but little from the overshadowing influence of reorganization, which seemed to occupy all minds.

The exhibit of the Ward's Natural Science Establishment of Rochester, N. Y., under the care of Mr. Charles H. Ward, was not only novel, as far as the American Dental Association was concerned, but was one, probably, never before brought together at a convention, or anywhere else. The time was too short, unfortunately, to give it that study that its great merits warranted; and it is to be hoped that it will in the future find a resting place in some one of our large educational institutions. The specimens consisted of jaws, crania, dentitions, and single teeth, fossil and recent, some of which were prepared in sections, and it embraced all the classes and order with many of the individual species of the vertebrates.

The lantern exhibit of Dr. I. N. Broomell was one of unusual interest, covering entirely new ground in the development of the teeth, in that it gave the macroscopical appearances of that process from the earliest period to the development of the first permanent molar. The dissections made showing the beginnings of the development of enamel and dentine were not only unique in character, but original in conception and execution.

Owing to important duties, the writer was unable to attend any of the sessions of the Southern Dental Association, but it is inferred from reports to have been equally satisfactory.

Both organizations, by a unanimous vote, concluded to meet in joint convention to complete a new organization. The Southern had decided it was not wise to abandon entirely the old organization, but that this should be continued as one of the branches of the national body. The members of the American, on the other hand, decided to abandon the Association and wind up its affairs. This was not accomplished without opposition, for it was felt by many that it would be better to continue it as the Eastern branch. It was further contended that it was impolitic, at this juncture, to dissolve this organization until the new one had gained a secure place in the interest of the profession. The majority controlled, and the American Dental Association ceased to exist on Friday, the 6th of August, 1897, at 12.30 P.M. The balance of the money in the treasury, after settling all indebtedness, was voted to the treasury of the Dental Protective Association, and the proceedings of many years, in the care of the secretary, were donated to the Army and Navy Medical Museum at Washington, D. C.

The final hour was one long to be remembered by those present. The American Dental Association had been a power in the dental profession for thirty-seven years. Its work had been, at times, unsatisfactory, but a true reading of its history will give to it a large share of the credit of whatever advance has been made in dental work during the period named. The remarks of the various speakers appropriately voiced this feeling, and they mourned the necessity of destroying this body that something more in accordance with changing circumstances might live.

The joint convention met at the Hygeia Hotel, Dr. Rich, of Washington, D. C., presiding. In a very brief period a constitution was adopted and a name given to the new organization, that of "The National Dental Association." This was so rapidly accomplished that those present were given but limited time to consider the main question before it was adopted. Then it was recalled that the laws prepared for its government had not even been read, although published in pamphlet form. The reading finally took place, but resulted in no change, and the convention entered into the election of officers, resulting in the choice of Dr. Thomas Fillebrown, of Boston, as president for the ensuing year. The other officers were divided between the two original associations. That this organization could be accomplished without friction was not expected, but through the skilful management of the Joint Committee there was no opposition manifested until an effort was made to change the name from that selected to one more definite. The discussion aroused some feeling, but it was finally left unchanged, the matter going over to the next session of this body, to be held at Omaha, Neb., in 1898.

This, in brief, is the history of the hour; for a more detailed account readers are referred to the report to be published in this journal.

The question which concerns all dentists of the United States is, What is to be the outcome of the change? Time alone can answer this; but it is quite apparent that the element of permanency does not appear to exist in the laws prepared for its government. These are decidedly overweighted, and it will be exceedingly difficult to govern under them. It is imagined the first business next year will be an effort to eliminate at least one-third of this organic law, for this amount might well be set aside without injury. The difficulties met with by the committee are fully appreciated, and the necessity understood for a speedy adoption of this instrument to avoid protracted discussion; but it must be regarded as hasty legislation, and this invariably means subsequent regret. The proper course, in the judgment of the writer, would have been to have made a short constitution and an equally brief series of by-laws, and, these adopted, the accretions could safely be left for experience to develop.

The period fixed for the annual meeting is a very questionable change. It will be practically impossible for dentists of the Eastern cities to meet at the time proposed. September means to a majority of these resumption of practice at the close of a protracted holiday, and they will view with little favor an extension to make a lengthy journey before recommencing professional labor. It is very possible that other sections may find this a convenient period, and it is anticipated that the first meeting at Omaha will have a large Western attendance, but, for the reason given, not many from Eastern sections.

The writer of this was an enthusiastic advocate of the union of the two principal associations of dentists in North America. It was, in his opinion, a waste of energy to distribute and weaken effort by the methods previously adopted. Hence this meeting was looked forward to as the opening of a new era in dentistry, in which some of the effete old would be eliminated and changes appropriate to the present period be installed as part of the new organization. That the majority clung tenaciously to the old was in accordance with the conservative feeling always present and not easily overcome; but it is, nevertheless, a great disappointment that the organic law contains not a single new feature. It fails to provide for the future; hence, in accordance with all past experience, it will not meet the demands of the coming generations.

The writer of this gave in his opening address the changes which, in his opinion, were necessary for the fuller development of the dental profession in this country, but none of these suggestions were considered of sufficient importance to be engrafted upon the organic law, and it is hopeless to expect these, either in whole or in part, will be adopted in the near future. One suggestion, it would seem, might have been consistently incorporated, that of providing a fund to assist in the investigation of unsolved problems.

In view of all the facts connected with this union meeting, it must sadly be acknowledged that not one step in advance has been made. The American Dental Association has passed into history and in its place a nominal union of the South with the North. It is to be hoped that the next meeting will make this a reality, but until this is accomplished we must regard the situation as entirely temporary and with nothing absolutely settled.

